# Canine Transforming Growth Factor-β Receptor 2-Ig: A Potential Candidate Biologic for Melanoma Treatment That Reverses Transforming Growth Factor-β1 Immunosuppression

**DOI:** 10.3389/fvets.2021.656715

**Published:** 2021-06-14

**Authors:** Hiroto Takeuchi, Satoru Konnai, Naoya Maekawa, Satoshi Takagi, Hiroshi Ohta, Noboru Sasaki, Sangho Kim, Tomohiro Okagawa, Yasuhiko Suzuki, Shiro Murata, Kazuhiko Ohashi

**Affiliations:** ^1^Department of Disease Control, Faculty of Veterinary Medicine, Hokkaido University, Sapporo, Japan; ^2^Department of Advanced Pharmaceutics, Faculty of Veterinary Medicine, Hokkaido University, Sapporo, Japan; ^3^Department of Clinical Sciences, Faculty of Veterinary Medicine, Hokkaido University, Sapporo, Japan; ^4^Department of Veterinary Surgery, School of Veterinary Medicine, Azabu University, Sagamihara, Japan; ^5^Research Center for Zoonosis Control, Hokkaido University, Sapporo, Japan; ^6^Global Station for Zoonosis Control, Global Institution for Collaborative Research and Education, Hokkaido University, Sapporo, Japan

**Keywords:** canine, melanoma, immunosuppression, TGF-β1, biologic, cancer immunotherapy

## Abstract

Cancer cells can evade host immune systems *via* multiple mechanisms. Transforming growth factor beta 1 (TGF-β1) is an immunosuppressive cytokine that induces regulatory T cell (Tregs) differentiation and is involved in immune evasion mechanisms in cancer. The inhibition of the TGF-β1 signaling pathway can suppress cancer progression and metastasis through the modulation of anticancer immune responses. However, to best of our knowledge, no implementation of treatments targeting TGF-β1 has been reported in dog cancers. This study aimed to examine whether TGF-β1 is upregulated in canine cancers. We measured TGF-β1 concentrations in culture supernatants of canine melanoma cell lines and in serum samples from dogs with oral malignant melanoma. TGF-β1 production was observed in several cell lines, and serum TGF-β1 levels were elevated in dogs with oral malignant melanoma. Interestingly, the addition of recombinant TGF-β1 to canine peripheral blood mononuclear cell cultures decreased Th1 cytokine production and increased differentiation of CD4^+^CD25^+^Foxp3^+^ lymphocytes, suggesting that TGF-β1 is immunosuppressive in canine immune systems. We developed a decoy receptor for TGF-β, namely TGF-βRII-Ig, by identifying an open reading frame of the canine *TGFBR2* gene. TGF-βRII-Ig was prepared as a recombinant fusion protein of the extracellular region of canine TGF-βRII and the Fc region of canine IgG-B. As expected, TGF-βRII-Ig bound to TGF-β1. In the presence of TGF-β1, the treatment with TGF-βRII-Ig increased Th1 cytokine production and decreased the differentiation of CD4^+^CD25^+^Foxp3^+^ lymphocytes. Our results suggest that TGF-βRII-Ig competitively inhibits the immunosuppressive effects of TGF-β1 and thereby activates immune responses. This study demonstrated the potential of TGF-βRII-Ig as a novel biologic for canine melanoma.

## Introduction

Transforming growth factor beta 1 (TGF-β1) is a multifunctional cytokine that is associated with cancer progression and suppression of immune response (1, 2). TGF-β1 directly inhibits cytotoxic T cell function by suppressing cell proliferation and the expression of cytotoxic genes (3, 4). TGF-β1 also inhibits the effector function of natural killer cells (5), contributing to immune evasion of cancer cells (6). In addition, TGF-β1 indirectly mediates immunosuppression by inducing regulatory T cells (Tregs) from peripheral naive T cells (7). Tregs inhibit interleukin (IL)-2 competitively through the increased expression of the IL-2 receptor, CD25, and suppress T cell function by producing immunosuppressive cytokines such as IL-10 and TGF-β1 (8). Therefore, TGF-β1 is considered a critical suppressor of Th1 responses in normal and diseased conditions, including cancers.

In a mouse model of metastatic breast cancer, TGF-β antagonism by an anti-TGF-β antibody resulted in significant enhancement of antitumor immune responses mainly mediated by CD8^+^ T cells (9). In a human phase I clinical trial, an anti-TGF-β antibody (GC1008) induced a partial response in one patient and stable disease in 6 of 28 patients with malignant melanoma and renal cell carcinoma (10). Similarly, galunisertib, a small-molecule inhibitor of transforming growth factor-β receptor 1, improved overall survival in patients with unresectable pancreatic cancer when combined with gemcitabine (11). These reports suggest that TGF-β1 inhibition is a possible strategy in developing immunotherapy against cancers.

In canine oncology, little information is available on the association of anticancer immunity with the TGF-β pathway. A previous study found the mean plasma TGF-β1 levels in tumor-bearing dogs were significantly higher than those in healthy controls (12). Canine transmissible venereal tumor, a unique, contagious cancer in canids, has been reported to produce TGF-β1 and suppress lymphokine-activated killer activity against tumor cells (13, 14). MiR-145, which is reported to regulate TGF-β, was downregulated in oral canine malignant melanoma tissue when compared with healthy oral mucosa tissue (15). In addition, previous studies have shown that canine mastocytoma and osteosarcoma cell lines produce TGF-β1 (16, 17) and TGF-β1 serum concentrations were higher in dogs with malignant perianal tumors (18). Furthermore, immunohistochemistry tests revealed that canine perivascular wall tumors and squamous cell cancers expressed TGF-β1 (19, 20). These studies suggest that the TGF-β1 pathway might be a common mechanism for evading immune responses exploited by canine cancers. However, there has been no report on TGF-β1 expression in other canine cancer types, and its immunosuppressive functions in dogs are still largely unknown.

In this study, we examined the potential of TGF-β1 as a therapeutic target in canine melanoma by confirming TGF-β1 production in melanoma cell lines and evaluating TGF-β1 serum concentrations in dogs with oral malignant melanoma (OMM). Next, we explored the immunosuppressive effects of TGF-β1 in canine peripheral blood mononuclear cell (PBMC) cultures. After that, we prepared, canine transforming growth factor-β receptor 2-Ig (TGF-βRII-Ig), which consists of an extracellular region of TGF-βRII and an Fc region of canine IgG-B, to investigate its efficacy as a candidate anticancer biologic, expecting it to inhibit the TGF-β1 pathway as a decoy receptor. Finally, the ability of TGF-βRII-Ig to bind TGF-β1 and its effect on immune response were investigated *in vitro*.

## Materials and Methods

### Canine Samples

The use of animal samples throughout this study was approved by the Institutional Animal Care and Use Committee (#15-0149), Faculty of Veterinary Medicine, Hokkaido University, which has been fully accredited by the Association for Assessment and Accreditation of Laboratory Animal Care International. Peripheral blood samples were obtained from healthy beagles (3–6 years old) kept at the Experimental Animal Facility, Faculty of Veterinary Medicine, Hokkaido University. Peripheral blood samples of dogs with OMM were obtained at the Veterinary Teaching Hospital, Faculty of Veterinary Medicine, Hokkaido University. Written informed consent was obtained from the owners for the participation of their animals in this study.

### Cell Cultures

Canine melanoma cell lines LMeC (21), CMeC (21), CMM-1 (22), CMM-2 (22) were cultured in RPMI 1640 medium (Sigma-Aldrich, St. Louis, MO, USA) supplemented with 10% inactivated fetal bovine serum (FBS; Thermo Fisher Scientific, Waltham, MA, USA), 2 mM L-glutamine (Thermo Fisher Scientific), 200 μg/mL streptomycin (Thermo Fisher Scientific), and 200 U/mL penicillin (Thermo Fisher Scientific) at 37°C, 5% CO_2_. Canine PBMCs were isolated from 10 mL of peripheral blood samples by density gradient centrifugation on Percoll medium (GE Healthcare, Little Chalfont, UK) and were cultured in RPMI 1640 medium supplemented with 10% inactivated FBS, 2 mM L-glutamine, 200 μg/mL streptomycin, and 200 U/mL penicillin at 37°C, 5% CO_2_. To activate PBMCs, 1 μg/mL Staphylococcal enterotoxin B from *Staphylococcus aureus* (SEB; Sigma-Aldrich) was added to the culture medium. ExpiCHO-S cells (Thermo Fisher Scientific) were cultured in ExpiCHO Expression Medium (Thermo Fisher Scientific) at 37°C, 8% CO_2_ on an orbital shaker.

### Measurement of TGF-β1 Concentration

The amino acid sequence of canine TGF-β1 is 100% identical to human TGF-β1. Thus, canine TGF-β1 concentration in cell culture supernatants and sera were measured by human TGF-β1 DuoSet ELISA (DY156, R&D systems, Minneapolis, MN). The absorbance was measured at 450 nm with an MTP-900Lab (CORONA ELECTRIC, Ibaraki, Japan).

### RNA Extraction and cDNA Synthesis

Canine white blood cells were prepared from lysed whole blood of a toy poodle and a papillon. Total RNA was extracted from PBMCs and white blood cells using TRI reagent (Molecular Research Center, Cincinnati, OH, USA). Residual genomic DNA was digested using DNaseI (Thermo Fisher Scientific), and cDNA was synthesized from 1 μg total RNA using PrimeScript Reverse Transcriptase (TaKaRa, Otsu, Japan) and oligo dT primer.

### Identification of Canine *TGFBR2* Gene

A primer pair for canine *TGFBR2* gene was designed based on the predicted dingo *TGFBR2* mRNA sequence registered in the GenBank database (XM_025461082.1). The open reading frame (ORF) of canine *TGFBR2* was amplified from beagle, toy poodle, and papillon cDNA by PCR using the primers 5′-TCG GTC TAT GAC GAG CAG C-3′ and 5′-GCT GCC TCT GTT CTT TGG TG-3′. The amplicon was purified using the FastGene gel/PCR extraction kit (Nippon Genetics, Tokyo, Japan) and cloned into T-Vector pMD20 (TaKaRa) using TA-cloning. The nucleotide sequence was analyzed using the GenomeLab GeXP Genetic Analysis System (SCIEX, Framingham, MA, USA), and the identified ORF was translated and aligned using BioEdit software (23). Unrooted neighbor-joining phylogenetic trees were constructed using Mega version 7 software (24, 25).

### Expression and Purification of Canine TGF-βRII-Ig and Control IgG-B

Recombinant canine TGF-βRII was expressed as an Fc-fusion protein (TGF-βRII-Ig) in a mammalian-cell based transient expression system. To predict the signal peptide sequence and transmembrane region, the deduced amino acid sequence of canine TGF-βRII was analyzed using the prediction tools: SignalP-5.0 Server (http://www.cbs.dtu.dk/services/SignalP/) for signal peptide prediction and TMHMM Server v. 2.0 (http://www.cbs.dtu.dk/services/TMHMM-2.0/) for transmembrane region prediction. The gene sequence encoding the predicted signal peptide and the extracellular region of canine TGF-βRII that was fused to the Fc region of canine IgG-B (AF354265.1) were designed, codon-optimized, and synthesized with an *Asc*I/*ASiS*I restriction site (GenScript, Piscataway, NJ, USA). The gene sequence was cloned into the expression vector pDC62c5-U533 (kindly provided by Prof. Yasuhiko Suzuki, Hokkaido University) using *Asc*I (New England Biolabs, Ipswich, MA, USA) and *AsiS*I (New England Biolabs), and the plasmid was purified using NucleoBond Xtra Midi (TaKaRa). To prepare a negative control for TGF-βRII-Ig, the expression vector that only encodes the canine IgG-B Fc region was similarly constructed using the signal peptide of canine antibody light chain (26). The expression plasmids were transfected into ExpiCHO cells using the ExpiFectamine CHO Transfection Kit (Thermo Fisher Scientific) and the expressed proteins were purified using Ab-Capcher ExTra (ProteNova, Kagawa, Japan). The buffer was replaced with phosphate-buffered saline (PBS; FUJIFILM Wako Pure Chemical, Osaka, Japan) using PD MidiTrap G25 (Cytiva, Tokyo, Japan). The concentration of the expressed proteins was measured using the Pierce BCA Protein Assay Kit (Thermo Fisher Scientific). Sodium dodecyl sulfate polyacrylamide gel electrophoresis (SDS-PAGE) was performed using 2 × Laemmli Sample Buffer (Bio-Rad, Hercules, CA, USA) containing 2-mercaptoethanol in reducing and non-reducing conditions (without 2-mercaptoethanol). The samples were incubated at 96°C for 5 min and separated by electrophoresis using SuperSep Ace (FUJIFILM Wako Pure Chemical). Precision Plus Protein All Blue Standards (Bio-Rad) was used as a protein standard. The gel was stained using the Quick-CBB kit (FUJIFILM Wako Pure Chemical).

### Detection of TGF-βRII-Ig Binding to TGF-β1

The binding of TGF-βRII-Ig to TGF-β1 was evaluated by ELISA. A 96-well ELISA microplate (SUMITOMO BAKELITE, Akita, Japan) was coated with 10 μg/mL of TGF-βRII-Ig or Control IgG-B diluted in PBS at 37°C overnight. After washing with PBS containing 0.05% Tween20 (KANTO KAGAKU, Tokyo, Japan), the plate was blocked with Super Block (Thermo Fisher Scientific) at 37°C for 1 h. Recombinant human TGF-β1 (240-B/CF, R&D systems) was added to the plate at various concentrations (4-fold dilution from 8 to 0.125 ng/mL), followed by incubation at 37°C for 1 h. Then, 1 μg/mL biotinylated chicken anti-human TGF-β1 antibody (R&D systems) was added and incubated at 37°C for 1 h. The reaction was developed using peroxidase-conjugated NeutrAvidin protein (Thermo Fisher Scientific) and TMB One Component Substrate (Bethyl Laboratories, Montgomery, TX, USA) and then stopped with 0.18 M H_2_SO_4_. As a dilution buffer for recombinant TGF-β1, anti-TGF-β1, and NeutrAvidin, PBS containing 1% bovine serum albumin was used. The absorbance was measured at 450 nm with the MTP-900Lab (CORONA ELECTRIC).

### Measurement of Cytokine Production From Canine PBMCs

Canine PBMCs from healthy beagles were cultured with SEB at 1 μg/mL for 72 h. Recombinant human TGF-β1 (0.78 nM) alone or in combination with TGF-βRII-Ig (78 nM) were added to the medium. Control IgG-B was used as negative control for TGF-βRII-Ig. IL-2, interferon (IFN)-γ, and tumor necrosis factor (TNF)-α concentrations in the culture supernatants were measured by Canine IL-2 DuoSet ELISA (DY1815, R&D systems), Canine IFN-γ DuoSet ELISA (DY781B, R&D systems), and Canine TNF-α DuoSet ELISA (DY1507, R&D systems), respectively. The absorbance was measured at 450 nm with the MTP-900Lab (CORONA ELECTRIC).

### Flow Cytometric Analysis of CD4^+^CD25^+^Foxp3^+^ Lymphocytes

Cells were collected from PBMC cultures as described above and blocked with PBS containing 10% goat serum (Thermo Fisher Scientific) at 25°C for 20 min. Subsequently, 1 μg/mL of anti-canine CD4 mouse monoclonal antibody (R&D systems) and 1 μg/mL phycoerythrin (PE)-conjugated anti-canine CD25 mouse monoclonal antibody (Thermo Fisher Scientific) were added and incubated at 25°C for 30 min. The dead cells were stained using Fixable Viability Dye eFluor780 (Thermo Fisher Scientific). Anti-canine CD4 was labeled with Alexa Fluor 488 using Zenon Mouse IgG2b Labeling Kits (Thermo Fisher Scientific). PE-labeled mouse IgG1 (Thermo Fisher Scientific) was used as an isotype control antibody for CD25 staining. The cells were washed twice with PBS, and then fixed using FOXP3 Fix/Perm Buffer (BioLegend, San Diego, CA, USA) and permeabilized using FOXP3 Perm Buffer (BioLegend). In the permeabilization buffer, 2% normal rat serum (Sigma-Aldrich) was added to block non-specific reactions. The cells were stained with 10 μg/mL of allophycocyanin-conjugated anti-canine Foxp3 rat monoclonal antibody (Thermo Fisher Scientific) at 4°C for 30 min. Allophycocyanin-labeled rat IgG2a kappa chain isotype control (Thermo Fisher Scientific) was used as a negative control. The cells were washed twice with FOXP3 Perm Buffer and analyzed using a FACS Verse flow cytometer (BD Biosciences, San Jose, CA, USA).

### Statistical Analysis

One-way ANOVA with Tukey's *post-hoc* test or Dunnett's *post-hoc* test were used for multiple comparison. The Wilcoxon singed-rank sum test was used to compare the paired samples. The Mann-Whitney *U*-test was used for comparison of two groups. A *p*-value <0.05 was considered statistically significant. JMP 14 (SAS Institute, Cary, NC, USA) was used for all statistical analysis.

### Nucleotide Sequence Accession Numbers

The nucleotide sequence of the canine *TGFBR2* gene has been submitted to the DDBJ/EMBL-Bank/GenBank database under accession number LC600803.

## Results

### Production of TGF-β1 by Canine Melanoma Cell Lines

**Figure 1:**
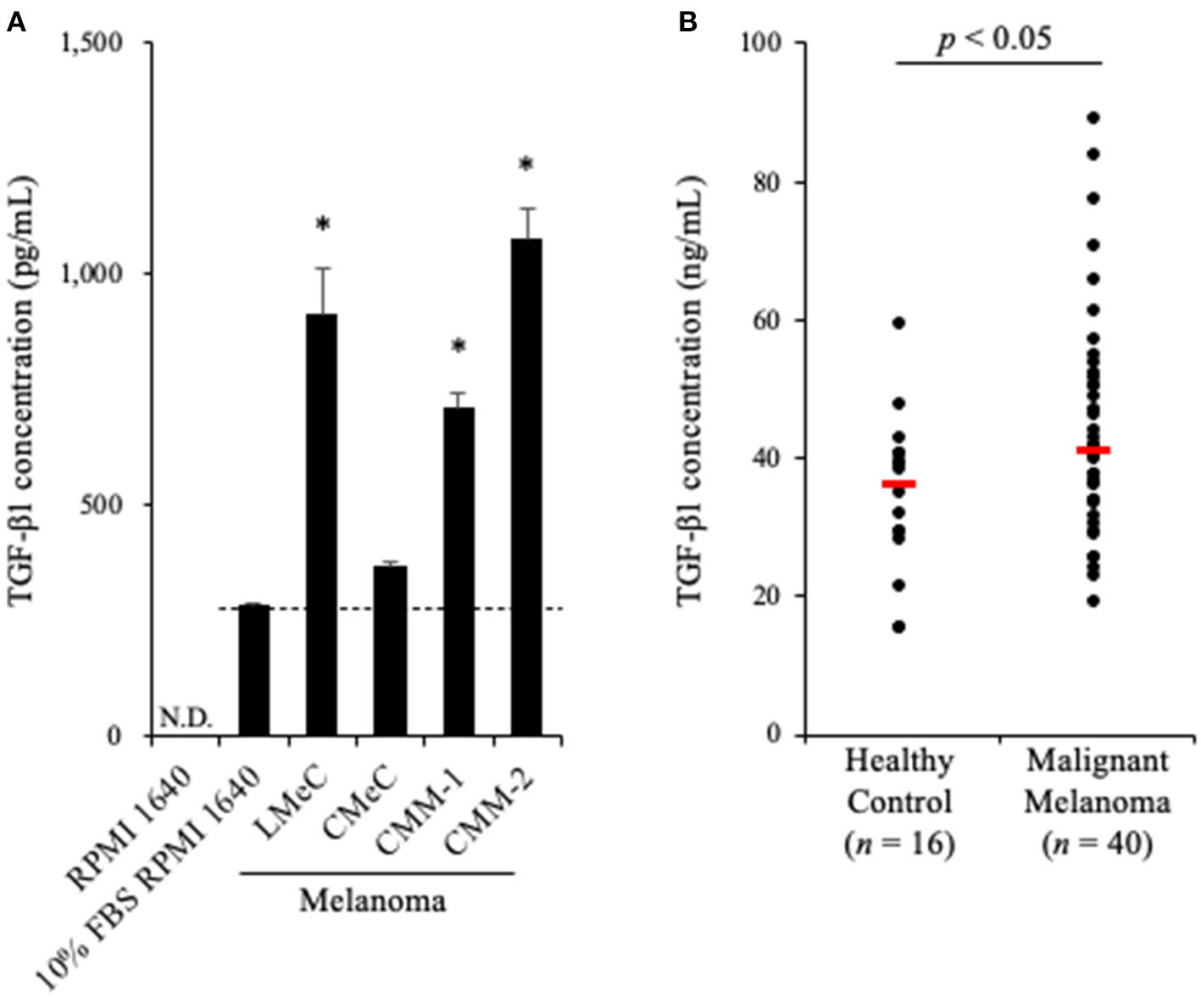
TGF-β1 expression in canine cancers. **(A)** TGF-β1 production by canine cancer cell lines. TGF-β1 concentration in 10% FBS RPMI 1640 is shown as the background (a dashed line). One-way ANOVA with *post-hoc* Dunnett's-test was used for statistical analysis under the assumption of equal variance between samples (*p <0.05 vs. 10% FBS RPMI 1640). **(B)** TGF-β1 serum levels in dogs with oral malignant melanoma. The red bar shows the median value. The Mann-Whitney *U*-test was used for statistical analysis.

To examine whether TGF-β1 is produced in a tumor microenvironment, we measured TGF-β1 concentrations in culture supernatants of canine melanoma cell lines. Melanoma cell lines were seeded into 6-well cell culture plate at a density of 2.0 ×10^5^ cells in 2 mL medium. After 48 h, when the cells were 60–80% confluent, the cell culture supernatant was collected. The cell culture was prepared in triplicate. Since FBS was expected to contain bovine TGF-β1, the mature amino acid sequence, which is 100% identical to canine and human TGF-β1, TGF-β1 concentration in RPMI 1640 medium supplemented with 10% FBS was measured as a background control. Of the four cell lines, LMeC, CMM-1, CMM-2 had significantly higher TGF-β1 concentrations in the culture supernatant than in the background (Figure 1A). These results confirmed that canine melanoma cells can produce TGF-β1.

### TGF-β1 Serum Levels in Dogs With OMM

Since we proved that canine melanoma cells produce TGF-β1, we hypothesized that the TGF-β1 serum levels in dogs with melanoma are upregulated. To examine this, serum samples were obtained from dogs with metastatic OMM (*n* = 40, Supplementary Table 1) and healthy beagles (*n* = 16), and the TGF-β1 serum levels were measured by ELISA. As expected, elevated TGF-β1 serum levels in dogs with metastatic OMM was observed (median, 36.4 ng/mL in healthy control vs. 41.1 ng/mL in metastatic OMM; Figure 1B). The range of TGF-β1 serum levels was 15.2–59.2 ng/mL in healthy control and 18.9–88.8 ng/mL in metastatic OMM. These results support the potential of targeting TGF-β1 as a melanoma treatment.

### Effects of TGF-β1 on Cytokine Production and Tregs Induction in Canine PBMC Cultures

**Figure 2:**
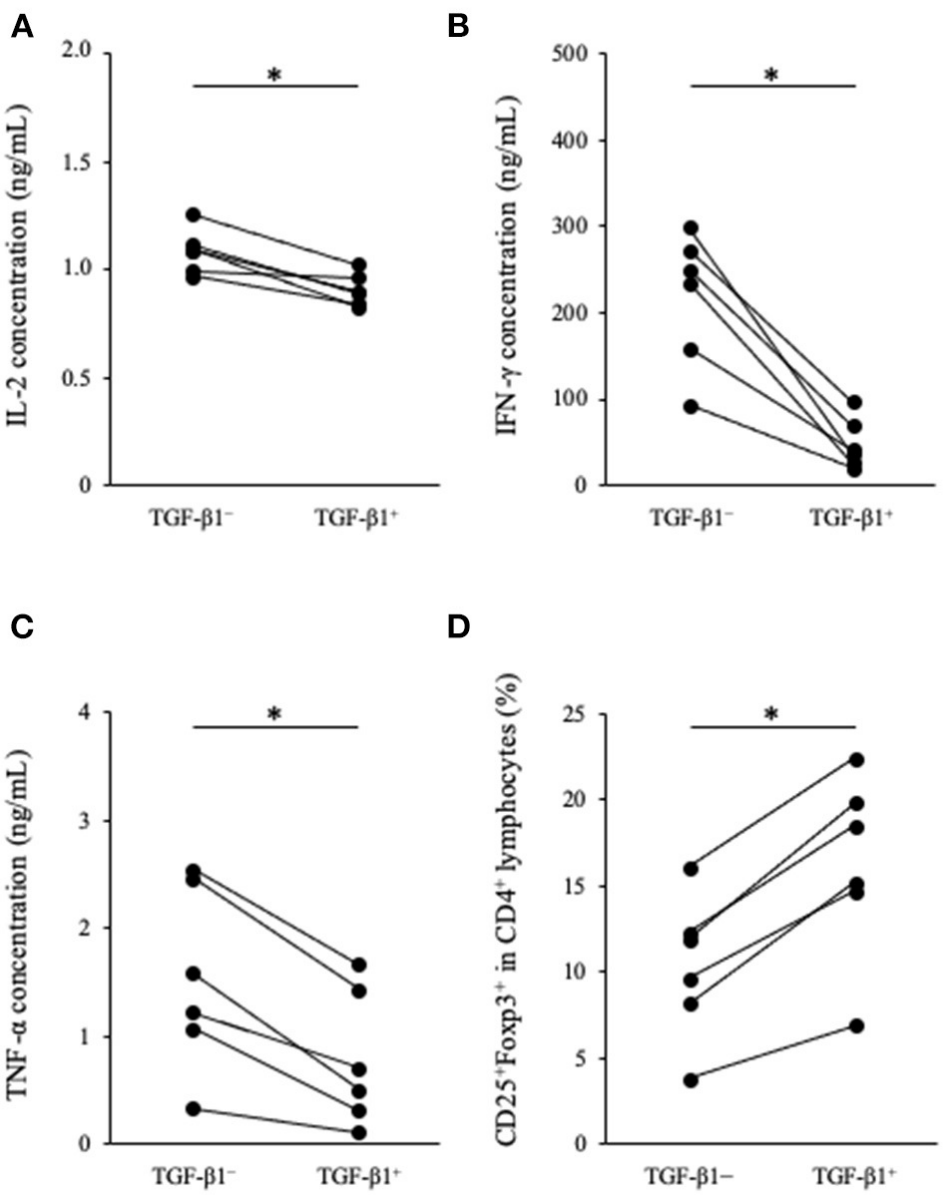
Effect of TGF-β1 on Th1 cytokine production and Tregs differentiation in PBMC cultures. **(A)** IL-2, **(B)** IFN-γ, and **(C)** TNF-α concentrations in cell culture supernatant were measured by ELISA. **(D)** The percentage of CD25^+^Foxp3^+^ cells among CD4^+^ lymphocytes was measured by flow cytometry. The Wilcoxon signed-rank sum test was used for statistical analysis (*n* = 6, *p <0.05).

To analyze the immunosuppressive effects of TGF-β1, we performed cytokine production assay in PBMCs cultures. PBMCs obtained from healthy beagles (*n* = 6) were seeded into 48-well cell culture plate at a density of 2.0 ×10^6^ cells in 1 mL medium and cultured for 3 days in the presence of TGF-β1. Th1 cytokine concentrations in the culture supernatant were measured by ELISA. IL-2, IFN-γ, and TNF-α concentrations were significantly decreased in the presence of TGF-β1 (Figures 2A–C). In addition, we tested the effects of TGF-β1 on the differentiation of CD4^+^CD25^+^Foxp3^+^ lymphocytes. These lymphocytes are considered a regulatory T cell subset in dogs and involved with immune response suppression (27). Flow cytometric analysis using 1 ×10^5^ cells per each condition was performed at the day of sample collection and revealed that the addition of TGF-β1 significantly increased the percentage of CD25^+^Foxp3^+^ cells among CD4^+^ lymphocytes (Figure 2D). The gating strategy is shown in Supplementary Figure 1.

### Identification of Canine *TGFBR2* Gene

**Figure 3:**
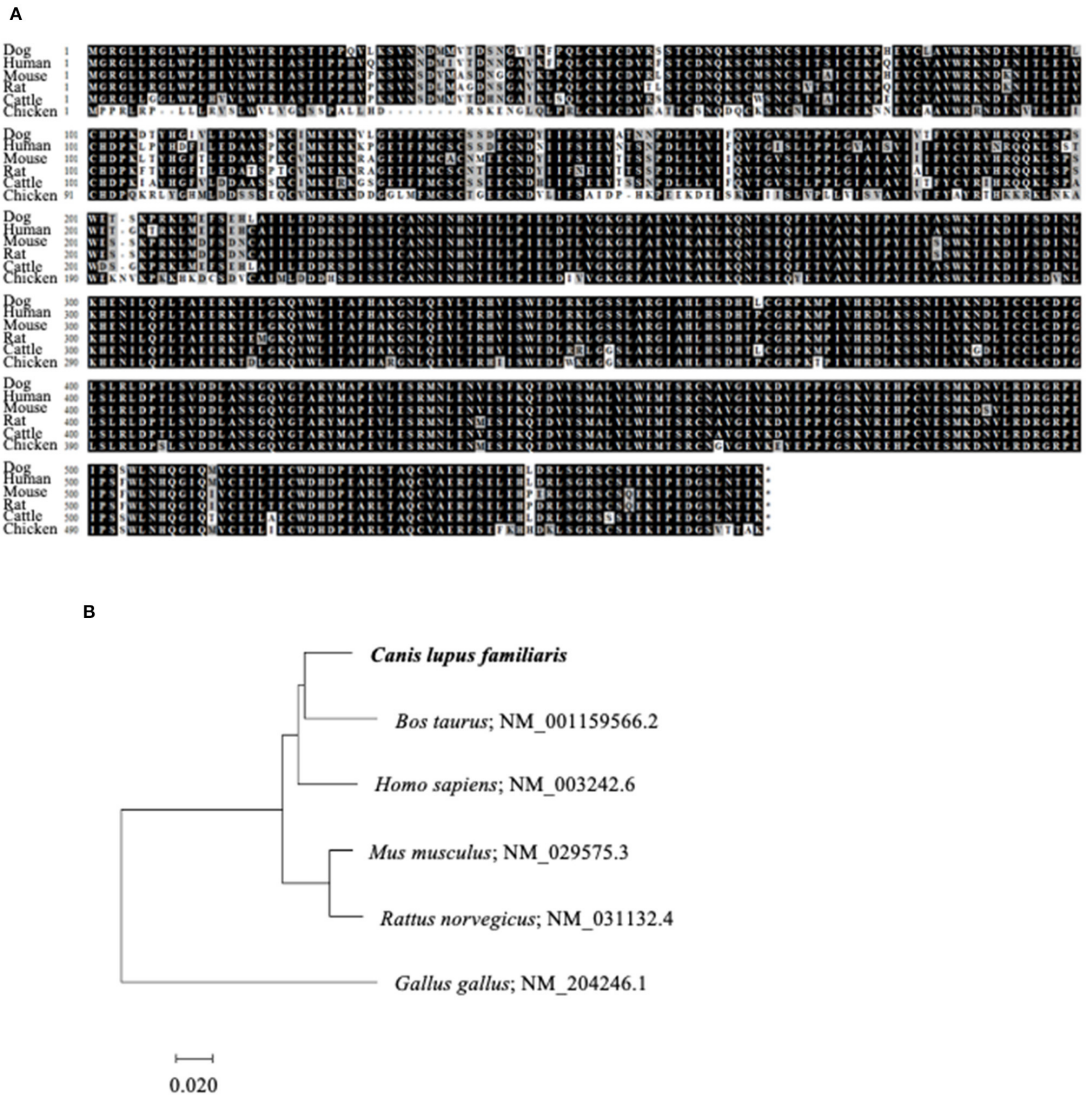
Identification of canine TGFBR2 gene. **(A)** Multiple alignment of TGF-βRII. The deduced amino acid sequence of canine *TGFBR2* gene was aligned with those of other species. **(B)** The phylogenetic tree of canine TGF-βRII in relation to those of other species. GenBank accession numbers were listed beside the scientific names. The scale bar indicates the branch length.

To reverse the immunosuppression by TGF-β1 in the tumor microenvironment, we attempted to develop a decoy receptor that competitively binds to TGF-β1. Among TGF-β1 receptors, TGF-βRII acts as a direct binding partner to TGF-β1 (28, 29). However, the *TGFBR2* gene, which encodes TGF-βRII, had not been identified in dogs. Therefore, we firstly identified the canine *TGFBR2* gene using cDNA from a beagle, a toy poodle, and a papillon. The ORF of canine *TGFBR2* was 1,704 bp in length and was identical among these three dog breeds. The deduced amino acid sequences of canine TGF-βRII showed high homology with those of other animals that were registered in the GenBank database (Figure 3A). The phylogenetic tree was inferred in relation to the deduced TGF-βRII amino acid sequences of other animals. Canine TGF-βRII formed a cluster with cattle and human TGF-βRII and was relatively distant to the rodent and chicken homologs (Figure 3B). Taken together, it is suggested that canine TGF-βRII has a similar function to TGF-βRII in other animals.

### Preparation of TGF-βRII-Ig and Control IgG-B

**Figure 4:**
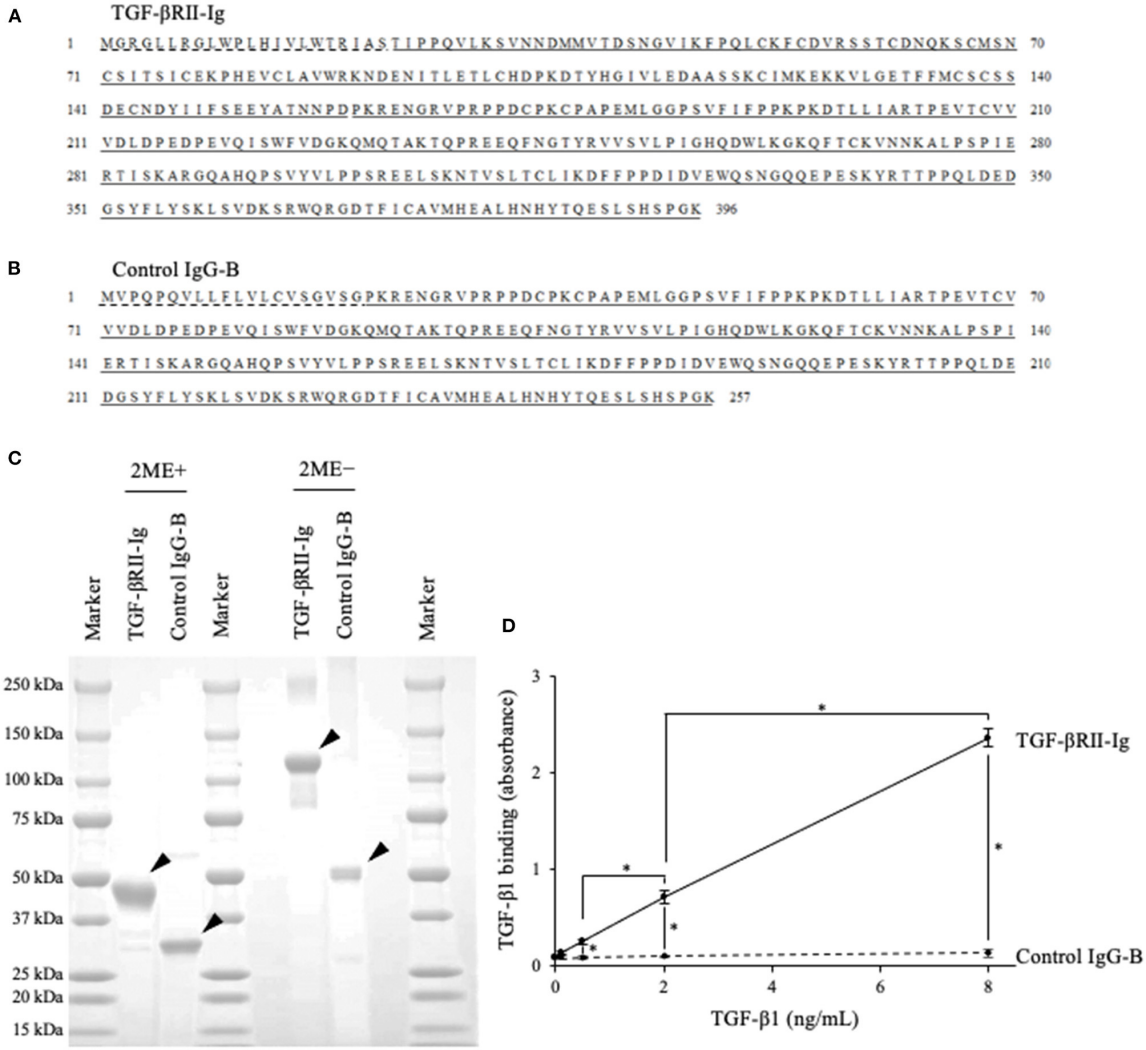
Preparation of recombinant TGF-βRII-Ig and Control IgG-B. **(A)** The amino acid sequences of TGF-βRII-Ig. The predicted signal peptide of TGF-βRII, extracellular region, and canine IgG-B Fc region are marked with a dashed line, underline, and double underline, respectively. **(B)** The amino acid sequences of Control IgG-B. The signal peptide of the canine antibody light chain and canine IgG-B Fc region are marked with a dashed line and double underline, respectively. **(C)** SDS-PAGE of the purified Ig proteins. Analysis in reducing (left) and non-reducing (right) conditions. Arrowheads indicate the protein bands with expected molecular weights. **(D)** Binding of TGF-βRII-Ig to TGF-β1. Each dot represents the average of absorbance obtained from three independent experiment. Error bars represent the standard deviation. One-way ANOVA with *post-hoc* Tukey's-test was used for statistical analysis (*p <0.05).

Based on the obtained sequence for the canine *TGFBR2* gene, we designed TGF-βRII-Ig, a Fc-fusion protein of the TGF-βRII extracellular region, and its control Ig protein, Control IgG-B (Figures 4A,B). Using a transient expression system, the Ig proteins were expressed and purified from the culture supernatant by affinity chromatography using a protein A derivative. Then, the TGF-βRII-Ig and Control IgG-B purities were analyzed by SDS-PAGE. The molecular weights of TGF-βRII-Ig and Control IgG-B were estimated at ~45 and 30 kDa, respectively, in reducing condition (Figure 4C). The hinge region of canine IgG-B Fc contains two cystine residues that would lead to the formation of a parallel dimer. Expectedly, the Ig proteins migrated at a higher molecular weight in non-reducing conditions, suggesting a homodimer formation with interchain disulfide bonds (Figure 4C).

### Binding of TGF-βRII-Ig to TGF-β1

To examine the binding of TGF-βRII-Ig to TGF-β1, an ELISA was developed using recombinant TGF-β1 and anti-TGF-β1 antibody. Various TGF-β1 concentrations were captured on a TGF-βRII-Ig-coated microwell plate, and the TGF-β1 binding was detected using anti-TGF-β1 antibody. As expected, the TGF-β1 binding was observed in a concentration-dependent manner, whereas no increase in the absorbance was observed in a microwell plate coated with Control IgG-B. These results indicated that TGF-βRII-Ig specifically binds to TGF-β1 (Figure 4D).

### Effects of TGF-βRII-Ig on Cytokine Production and Tregs Induction in Canine PBMC Cultures in the Presence of TGF-β1

**Figure 5:**
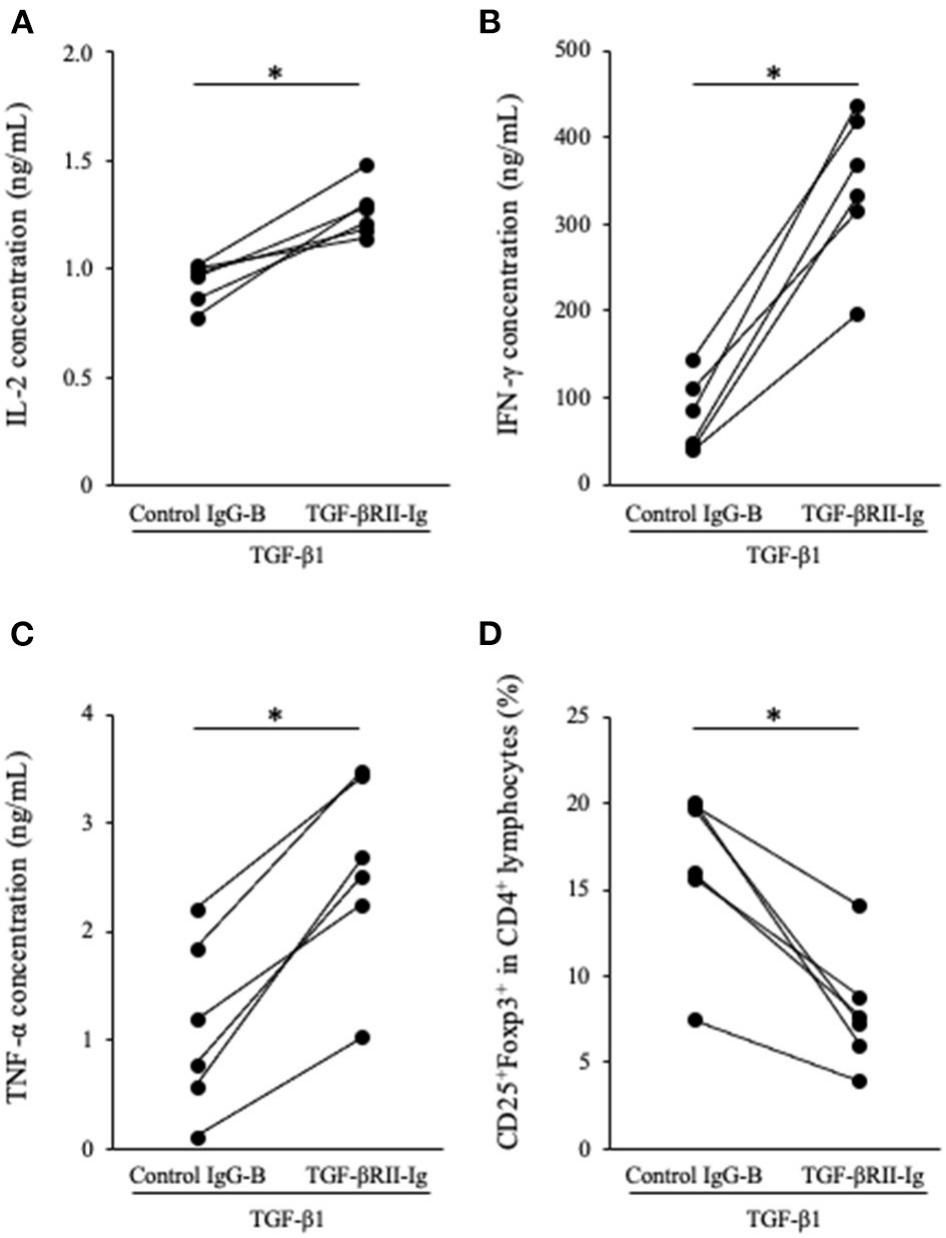
Effect of TGF-βRII-Ig on Th1 cytokine production and Tregs differentiation of in PBMC cultures in the presence of TGF-β1. **(A)** IL-2, **(B)** IFN-γ, and **(C)** TNF-α in cell culture supernatants were measured by ELISA. **(D)** The percentage of CD25^+^Foxp3^+^ cells among CD4^+^ lymphocytes was calculated by flow cytometry. The Wilcoxon signed-rank sum test was used for statistical analysis (*n* = 6, *p <0.05).

To analyze the function of TGF-βRII-Ig, TGF-βRII-Ig was added to the PBMC cultures in the presence of TGF-β1. TGF-βRII-Ig was added at a 100:1 molar ratio to exogenous TGF-β1 to block the effect of TGF-β1 completely. Cytokine production and Tregs induction were analyzed as described above. IL-2, IFN-γ, and TNF-α production in culture supernatants were significantly increased by treatment with TGF-βRII-Ig compared with Control IgG-B (Figures 5A–C). In addition, the TGF-βRII-Ig treatment significantly decreased the percentage of CD25^+^Foxp3^+^ cells among CD4^+^ lymphocytes (Figure 5D).

## Discussion

In this study, we confirmed the production of TGF-β1 by the specific melanoma cell lines tested, possibly contributing to the elevation of TGF-β1 serum levels in melanoma dogs. The addition of TGF-β1 decreased Th1 cytokine production from PBMCs and induced Tregs differentiation, indicating its immunosuppressive effect. The decoy receptor TGF-βRII-Ig bound specifically to TGF-β1 and reversed cytokine production and Tregs differentiation induced by TGF-β1. These data strongly suggest that TGF-βRII-Ig competitively inhibits immunosuppressive effects of TGF-β1, resulting in the reversal of Th1 immune response and inhibition of Tregs differentiation. Hence, targeting TGF-β1 using TGF-βRII-Ig is a potential therapeutic strategy for canine melanoma treatment.

To our knowledge, this is the first report that observed TGF-β1 production in canine malignant melanoma cell lines and elevation of serum TGF-β1 in dogs with metastatic OMM. Since TGF-β1 is involved in morphological changes associated with cancer cell migration and metastasis (1), we studied dogs diagnosed with metastatic OMM presenting with lymph node metastasis or distant metastasis. Due to the limited access to clinical samples of non-metastatic OMM, we could not evaluate TGF-β1 serum levels in dogs with non-metastatic OMM. Some dogs with metastatic OMM had lower TGF-β1 serum levels than healthy control. Considering the different levels of TGF-β1 production from melanoma cell lines, it may reflect the different cancer phenotype. Because various cell types, including tumor-infiltrating lymphocytes, can produce TGF-β1 in a tumor microenvironment (30), the source of serum TGF-β1 in canine metastatic OMM remains unclear. Further studies that include immunohistochemistry analysis or single-cell RNA sequencing using tissue samples of OMM might be beneficial to address this issue. Nonetheless, TGF-β1serum levels and expression in cancer tissues should be analyzed in various types of cancers to explore the clinical indications of TGF-βRII-Ig as a potential therapeutic agent.

In humans, higher TGF-β1 in plasma was associated with reduced overall survival in patients with locally advanced or metastatic pancreatic cancer (31). In addition, TGF-β1 plasma levels in lung carcinoma patients were correlated with the disease status at long-term follow-up after radiotherapy (32). Osteosarcoma patients with metastatic disease also had higher serum TGF-β than those without metastasis (33). Therefore, TGF-β1 serum levels could be used as a prognostic factor in human cancers. In dogs, the association between TGF-β1 serum levels and cancer prognosis remains unclear. Further studies involving samples coupled with clinical data are needed to examine whether TGF-β1 serum levels can be used as a prognostic marker in dogs with cancer.

Tregs are involved in the suppression of immune responses to foreign antigens, such as viruses (34), and immune evasion mechanisms in cancers (35). Interestingly, the percentage of Tregs in the peripheral blood of dogs with OMM and carcinoma was higher than that of healthy control dogs (36, 37). In addition, the increased number of intratumoral Tregs was related to shorter overall survival in dogs with OMM, oral squamous cell carcinoma, and pulmonary adenocarcinoma (38). Given that the present study confirms that serum TGF-β1 levels were higher in metastatic OMM dogs and that the addition of TGF-β1 induced Tregs differentiation, it is possible that TGF-β1 is responsible for Tregs induction in canine cancers, which leads to poor prognosis.

A limitation of this study is the lack of assessment of the immunostimulatory potential of TGF-βRII-Ig in the absence of TGF-β1. Since PBMC culture contains a certain amount of TGF-β1 derived from stimulated PBMC and FBS, the immunostimulatory potential of TGF-βRII-Ig might not be separated from its effects of reversing TGF-β1 immunosuppression.

In this study, we observed that TGF-β1 serum levels were elevated in dogs with metastatic OMM, and TGF-βRII-Ig successfully enhanced Th1 immune responses in PBMC cultures. The immunostimulatory effects of TGF-βRII-Ig should be further investigated in clinical studies including OMM dogs.

In conclusion, we confirmed that canine melanoma cells can produce TGF-β1 and TGF-β1 serum levels in dogs with melanoma are upregulated. In addition, TGF-β1 suppressed immune response in dogs. We successfully obtained TGF-βRII-Ig, which, as expected, reversed the TGF-β1 immunosuppression. Our results highlighted the potential of TGF-β1 as a target for canine cancer immunotherapy. Furthermore, the current findings support the further investigation of TGF-βRII-Ig as a candidate biologic enhancing anticancer immunity. The antitumor efficacy and safety of TGF-βRII-Ig should be evaluated in clinical studies targeting dogs with cancers as a single agent or in combination with existing treatment modalities.

## Data Availability Statement

The nucleotide sequence of the canine TGFBR2 gene has been submitted to the DDBJ/EMBL-Bank/GenBank database under accession number LC600803.

## Ethics Statement

The animal study was reviewed and approved by the use of animal samples throughout this study was approved by the Institutional Animal Care and Use Committee (#15-0149), Faculty of Veterinary Medicine, Hokkaido University, which has been fully accredited by the Association for Assessment and Accreditation of Laboratory Animal Care International. Peripheral blood samples were obtained from healthy beagles kept at the Experimental Animal Facility, Faculty of Veterinary Medicine, Hokkaido University. Peripheral blood samples of dogs with OMM were obtained at the Veterinary Teaching Hospital, Faculty of Veterinary Medicine, Hokkaido University. Written informed consent was obtained from the owners for the participation of their animals in this study.

## Author Contributions

SKo, SM, and KO designed and supervised the project. HT and NM performed the experiments. HT, SKo, NM, and TO analyzed the data. ST, HO, NS, SKi, and YS contributed reagents, materials, and analysis tools. HT, SKo, and NM prepared the manuscript. All authors reviewed and approved the manuscript.

## Conflict of Interest

The authors declare that the research was conducted in the absence of any commercial or financial relationships that could be construed as a potential conflict of interest.
