# Supplementary material for: Canine Transforming Growth Factor-β Receptor 2-Ig: A Potential Candidate Biologic for Melanoma Treatment That Reverses Transforming Growth Factor-β1 Immunosuppression
Source: Front Vet Sci. 2021 Jun 14;8:656715. doi: 10.3389/fvets.2021.656715 (PMC8236594; doi:10.3389/fvets.2021.656715)
Supplement: Supplementary file 1 [file Data_Sheet_1.PDF]

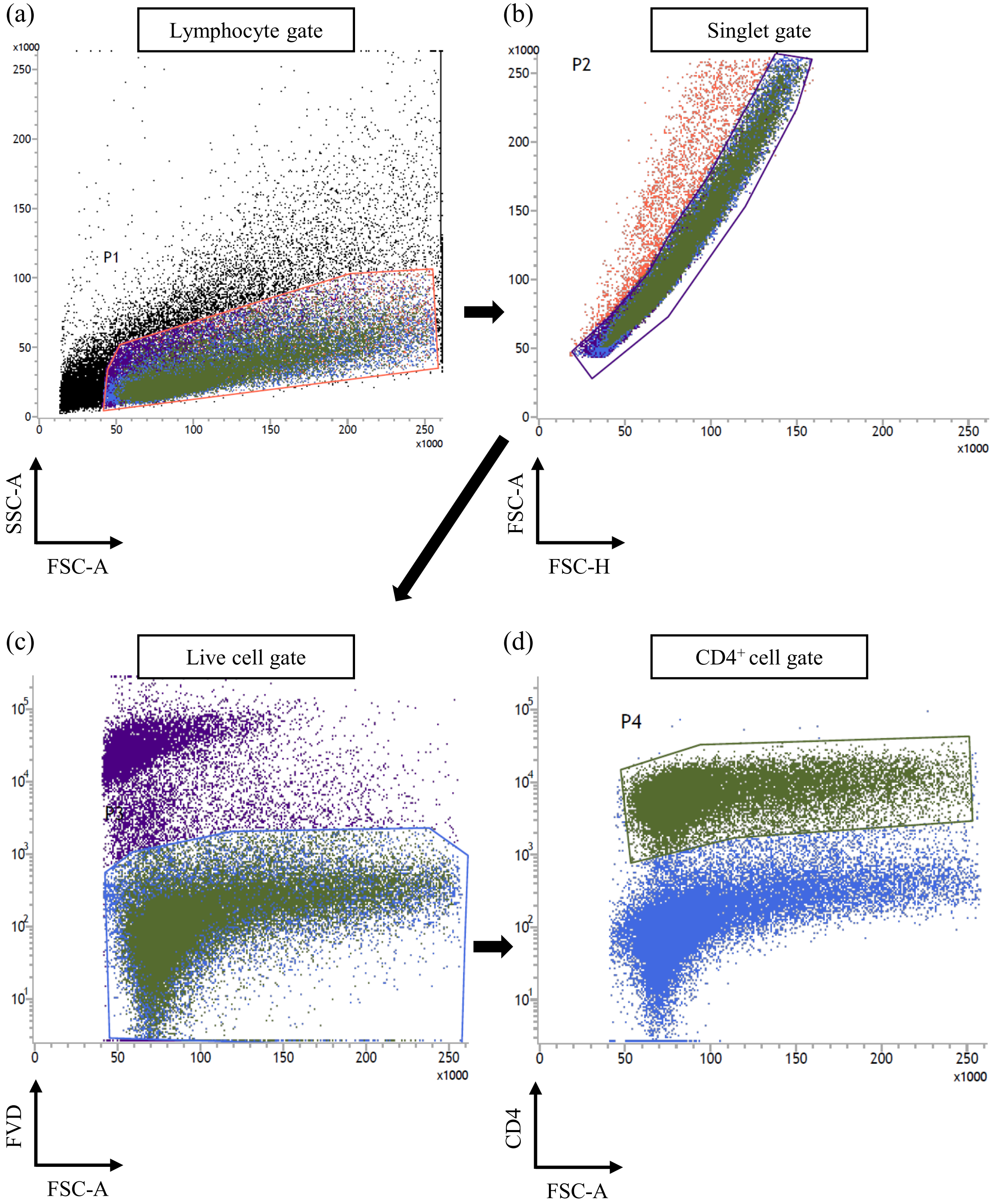

(a)
Lymphocyte gate
P1
SSC-A
FSC-A
(b)
Singlet gate
P2
FSC-A
FSC-H
(c)
Live cell gate
P3
FVD
FSC-A
(d)
CD4+ cell gate
P4
CD4
FSC-A

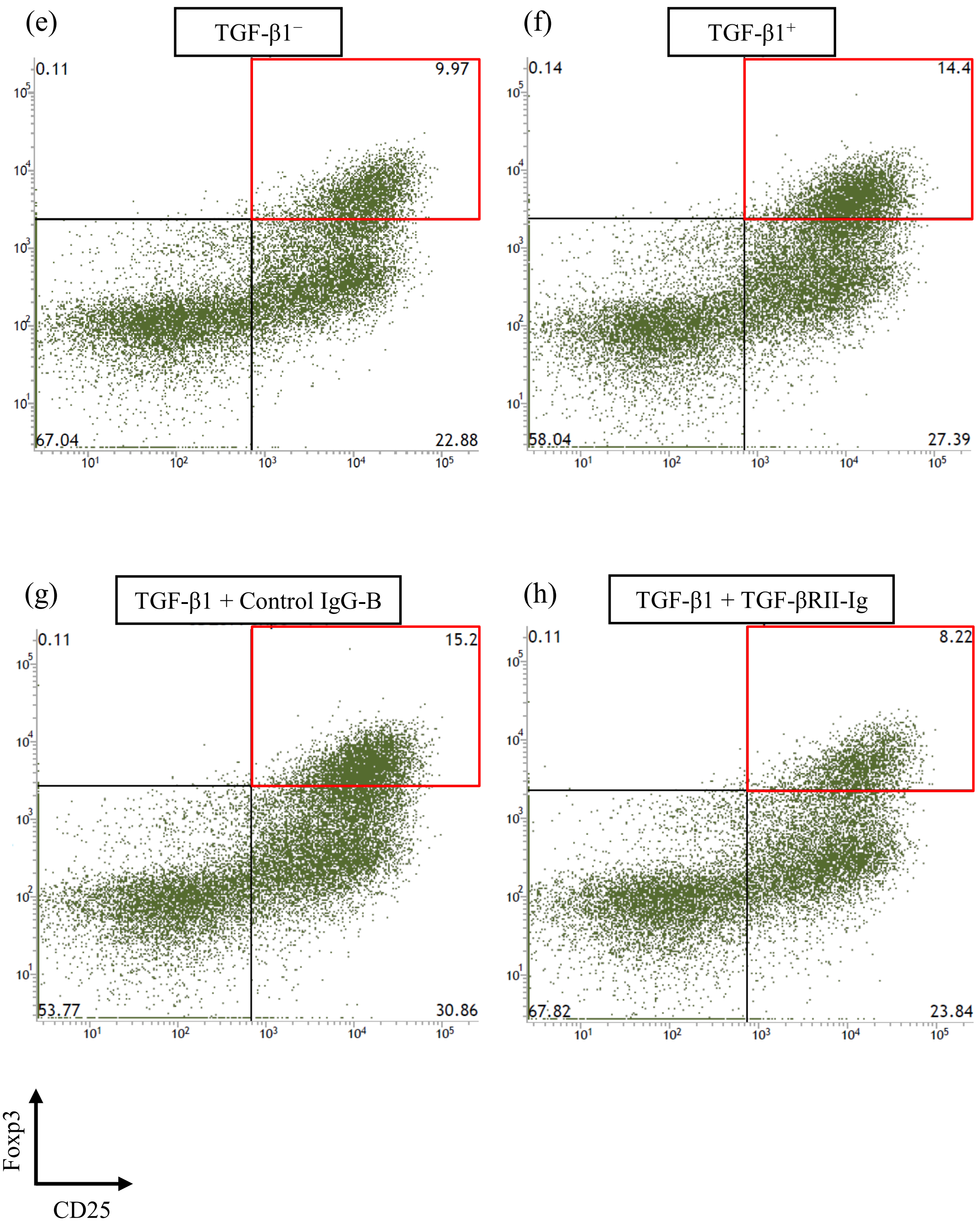

Supplemental Figure 1. The gating strategy for Tregs.

Total lymphocytes were first gated on a forward scatter (FSC)/side scatter (SSC) plot (a) and then gated on the singlet population (b). Cells were further gated to exclude dead cells by staining with FVD (c). Cells were then further gated for CD4 expression (d) and analyzed for CD25 and Foxp3 expression. The representative dot plots were shown in (e) TGF-β^{-}, (f) TGF-β^{+}, (g) Control IgG-B with TGF-β1, and (h) TGF-βRII-Ig with TGF-β1.

Supplementary Table 1. Characteristics of dogs with metastatic OMM.

Dog #	Breed	Sex	Age (year)	Concurrent disease	Stages of melanoma	TGF-β1 serum levels (ng/mL)
1	Pug	Male, castrated	11	None	IV	43.9
2	Miniature dachshund	Male, castrated	14	None	IV	52.0
3	Golden retriever	Male, castrated	10	None	IV	39.7
4	Miniature dachshund	Male, castrated	14	None	IV	77.2
5	Beagle	Female, spayed	11	None	IV	24.1
6	American cocker spaniel	Male, castrated	13	Chronic kidney disease, hypothyroidism, chronic pancreatitis	IV	48.5
7	Yorkshire terrier	Male	14	Inguinal hernia, intervertebral disk herniation	IV	50.2
8	Kaninchen dachshund	Female, spayed	13	None	IV	33.5
9	Miniature dachshund	Male	13	Hypothyroidism, megaesophagus, atopic dermatitis	IV	35.8
10	Pomeranian	Male	12	Polycystic kidney, dilated cardiomyopathy	IV	37.6

11	Mix	Female	12	None	IV	33.5
12	Pekingese	Male, castrated	8	Keratoconjunctivitis sicca	IV	54.7
13	Mix	Male, castrated	11	None	IV	29.0
14	Miniature dachshund	Male	13	None	IV	36.4
15	Miniature dachshund	Female	15	Pyometra	IV	51.2
16	Labrador retriever	Female	11	None	IV	56.9
17	Toy poodle	Female, spayed	13	Myxomatous mitral valve degeneration	IV	29.1
18	Miniature dachshund	Female, spayed	15	None	IV	40.1
19	Miniature dachshund	Male	14	None	IV	61.2
20	Labrador retriever	Male	12	None	IV	37.3
21	Toy poodle	Male	16	None	IV	25.4
22	Chihuahua	Female, spayed	13	Myxomatous mitral valve degeneration	IV	46.8
23	Welsh Corgi	Male, castrated	10	Pituitary macroadenoma, Cushing's disease	IV	50.2
24	Papillon	Male, castrated	14	None	IV	41.5

25	Flat-coated retriever	Female, spayed	8	None	IV	41.5
26	Golden retriever	Male, castrated	14	None	IV	25.2
27	Miniature dachshund	Male, castrated	14	Hydronephrosis	IV	30.2
28	Golden retriever	Male, castrated	12	None	IV	31.4
29	Pomeranian	Male	13	None	IV	53.6
30	Miniature dachshund	Female, spayed	10	None	IV	36.2
31	Miniature dachshund	Male, castrated	15	None	IV	70.5
32	Miniature dachshund	Male, castrated	14	None	IV	22.7
33	Mixed	Female, spayed	14	None	IV	33.3
34	Toy poodle	Female	13	None	IV	83.5
35	Mixed	Female, spayed	15	None	IV	45.9
36	Chihuahua	Male, castrated	13	Mitral insufficiency	IV	42.6
37	Miniature dachshund	Male, castrated	14	None	III	40.7
38	Shiba	Male, castrated	11	None	III	18.9

39	Miniature dachshund	Female	15	None	III	88.8
40	Miniature dachshund	Male	15	None	III	65.8
